# Association of muscle lipidomic profile with high-fat diet-induced insulin resistance across five mouse strains

**DOI:** 10.1038/s41598-017-14214-1

**Published:** 2017-10-24

**Authors:** Magdalene K. Montgomery, Simon H. J. Brown, Todd W. Mitchell, Adelle C. F. Coster, Gregory J. Cooney, Nigel Turner

**Affiliations:** 1 Department of Pharmacology, School of Medical Sciences, University of New South Wales, Sydney, NSW Australia; 2 Diabetes & Metabolism Division, Garvan Institute of Medical Research, Darlinghurst, NSW Australia; 3 School of Medicine, University of Wollongong, Wollongong, NSW Australia; 4 llawarra Health and Medical Research Institute, University of Wollongong, Wollongong, NSW Australia; 5 School of Mathematics and Statistics, University of New South Wales, Sydney, NSW Australia; 6 St Vincent’s Clinical School, University of New South Wales, Sydney, NSW Australia

## Abstract

Different mouse strains exhibit variation in their inherent propensities to develop metabolic disease. We recently showed that C57BL6, 129X1, DBA/2 and FVB/N mice are all susceptible to high-fat diet-induced glucose intolerance, while BALB/c mice are relatively protected, despite changes in many factors linked with insulin resistance. One parameter strongly linked with insulin resistance is ectopic lipid accumulation, especially metabolically active ceramides and diacylglycerols (DAG). This study examined diet-induced changes in the skeletal muscle lipidome across these five mouse strains. High-fat feeding increased total muscle triacylglycerol (TAG) content, with elevations in similar triacylglycerol species observed for all strains. There were also generally consistent changes across strains in the abundance of different phospholipid (PL) classes and the fatty acid profile of phospholipid molecular species, with the exception being a strain-specific difference in phospholipid species containing two polyunsaturated fatty acyl chains in BALB/c mice (i.e. a diet-induced decrease in the other four strains, but no change in BALB/c mice). In contrast to TAG and PL, the high-fat diet had a minor influence on DAG and ceramide species across all strains. These results suggest that widespread alterations in muscle lipids are unlikely a major contributors to the favourable metabolic profile of BALB/c mice and rather there is a relatively conserved high-fat diet response in muscle of most mouse strains.

## Introduction

Much of our insight into the role of specific genes in different disease states has been derived from studies in transgenic and knockout mouse models. However, with the increased use of genetically manipulated mice, it has become clear that the genetic background of the mice is a critical factor that needs to be carefully considered. For example, with respect to metabolic diseases, previous studies from our group and others have demonstrated that various mouse strains differ substantially in their metabolic profile and their susceptibility to develop lipid-induced obesity and insulin resistance^1–4^. Specifically, we showed that when five commonly used mouse strains (C57BL/6, 129X1/SvJ, BALB/c, DBA/2 and FVB/N) were exposed to a lard-based high fat diet (HFD), BL6, 129X1, DBA and FVB mice became glucose intolerant and insulin resistant to varying degrees, but the deterioration in glucose metabolism and insulin action was absent in BALB/c mice^4^.

Many factors have been suggested to be responsible for inducing defects in glucose homeostasis and insulin sensitivity, including inflammation, activation of stress pathways and ectopic lipid accumulation^5–7^. We recently showed that differential diet-induced changes in hepatic lipid composition was one factor that appeared to be partly responsible for the protection against lipid-induced glucose intolerance and insulin resistance observed in BALB/c mice^8^. Another tissue that plays a major role in whole-body insulin sensitivity is skeletal muscle, being one of the main sites of peripheral insulin-stimulated glucose uptake (as reviewed in^9^). There are strong associations between skeletal muscle lipid accumulation and insulin resistance in both humans and animals^10–13^, but the precise lipid species that are involved in impairing muscle insulin action are not fully resolved.

Given the disparate metabolic response of different mouse strains to the same dietary stimulus and the fact that HFD-fed BALB/c mice have preserved glucose homeostasis, in spite of excess adiposity and changes in other factors linked with insulin resistance^4^, we undertook detailed lipidomic profiling of skeletal muscle, to investigate if alterations in specific myocellular lipid species were correlated with insulin action across different strains of mice.

## Results

### Metabolic characterization

We have previously reported a comprehensive metabolic analysis of the mice used in this study^4^. Whereas all mouse strains exhibited a significant increase in whole-body fat mass, BALB/c mice were the only mouse strain that did not exhibit an increase in fasting blood glucose, fasting plasma insulin or area-under-the-curve (AUC) during an intraperitoneal glucose tolerance test, nor a deterioration of insulin sensitivity^4^. Taken together, the metabolic characterisation indicated that, in contrast to the other strains, BALB/c mice were largely spared from the HFD-induced deterioration of glucose tolerance and insulin sensitivity, despite a similar increase in whole-body adiposity^4^.

### Total abundance of various lipid classes in muscle and changes with high-fat feeding

Table 1 shows the total levels of various lipid classes in control and high-fat diet-fed mice, the number of lipid species in each class, as well as the amount of lipid species that were significantly increased or decreased by high-fat feeding. All five mouse strains exhibited a significant increase in TAG accumulation in muscle after high-fat feeding (Table 1). In contrast, total DAG, ceramide (Cer) and sphingomyelin (SM) levels remained unchanged, while total cholesterol ester (CE) increased significantly in 129X1, BALB/c and FVB/N mice. There was a small, but significant increase in total PL with HFD in 129X1 mice, while total PL abundance, as well as total PC levels, remained unchanged in the other strains. Minor increases in PE and PE-O (ether-linked PE) were observed in 129X1 and BALB/c mice, whereas PE-O showed a small decrease in DBA/2. PS abundance remained unchanged with HFD in all five mouse strains (Table 1).

**Table 1:** Lipid classes in muscle in CHOW- and HFD-fed mice and directional changes in lipid species with high-fat feeding.

Class	Abbr.	Species In Class	C57BL/6				129X1/SvJ				BALB/c				DBA/2				FVB/N			
			**CHOW**	**HFD**	**↓**	**↑**	**CHOW**	**HFD**	**↓**	**↑**	**CHOW**	**HFD**	**↓**	**↑**	**CHOW**	**HFD**	**↓**	**↑**	**CHOW**	**HFD**	**↓**	**↑**
Ceramide	CER	9	0.07 ± 0.01	0.07 ± 0.01	0	0	0.05 ± 0.01	0.06 ± 0.01	0	0	0.07 ± 0.01	0.07 ± 0.01	0	0	0.07 ± 0.01	0.07 ± 0.01	0	0	0.05 ± 0.01	0.06 ± 0.01	0	0
Sphingomyelin	SM	27	0.28 ± 0.03	0.28 ± 0.03	1	3	0.27 ± 0.01	0.30 ± 0.02	1	5	0.31 ± 0.02	0.31 ± 0.01	1	1	0.34 ± 0.03	0.29 ± 0.01	3	2	0.32 ± 0.01	0.30 ± 0.01	2	0
Diacylglycerol	DAG	14	1.55 ± 0.27	1.80 ± 0.42	0	0	1.91 ± 0.24	1.72 ± 0.13	0	0	2.12 ± 0.31	1.92 ± 0.22	0	0	2.07 ± 0.35	1.88 ± 0.15	0	0	1.43 ± 0.04	1.79 ± 0.11	0	0
Triacylglycerol	TAG	32	7.81 ± 1.70	**12.10 ± 2.82**	0	4	12.64 ± 1.72	**31.61 ± 1.78**	0	13	13.31 ± 1.27	**23.48 ± 2.28**	0	6	17.57 ± 2.04	**45.16 ± 6.08**	0	12	12.90 ± 0.67	**21.07 ± 1.58**	0	4
Cholesterolester	CE	9	0.08 ± 0.03	0.04 ± 0.01	0	0	0.04 ± 0.01	**0.14 ± 0.02**	0	3	0.02 ± 0.01	**0.06 ± 0.01**	0	1	0.07 ± 0.03	0.05 ± 0.01	0	0	0.06 ± 0.02	**0.10 ± 0.001**	0	0
Phospholipids	PL	96	14.75 ± 1.49	14.12 ± 0.35	15	15	12.64 ± 0.59	**13.96 ± 0.41**	6	18	14.94 ± 1.23	14.75 ± 0.28	15	12	15.97 ± 0.63	15.46 ± 0.49	15	11	13.02 ± 0.35	13.73 ± 0.35	7	15
Phosphatidyl choline	PC	35	11.27 ± 1.13	10.84 ± 0.26	8	7	9.97 ± 0.58	10.38 ± 0.32	5	7	12.08 ± 0.97	11.38 ± 0.24	11	6	12.11 ± 0.45	11.61 ± 0.37	7	4	9.96 ± 0.18	10.20 ± 0.29	6	5
Alkyl Phosphatidyl choline	PC(O)	13	0.28 ± 0.03	**0.31 ± 0.01**	2	2	0.26 ± 0.01	**0.31 ± 0.02**	0	2	0.33 ± 0.01	0.34 ± 0.01	2	2	0.40 ± 0.02	0.37 ± 0.01	2	2	0.22 ± 0.01	**0.27 ± 0.01**	1	1
Phosphatidyl ethanolamine	PE	26	3.15 ± 0.34	2.93 ± 0.13	4	6	2.39 ± 0.08	**3.26 ± 0.12**	1	5	2.55 ± 0.27	**3.02 ± 0.07**	2	3	3.51 ± 0.30	3.46 ± 0.09	4	3	2.74 ± 0.16	3.16 ± 0.17	0	6
Alkyl Phosphatidyl ethanolamine	PE(O)	16	0.53 ± 0.09	0.48 ± 0.03	1	0	0.39 ± 0.04	**0.70 ± 0.05**	0	3	0.34 ± 0.08	**0.51 ± 0.03**	0	0	0.66 ± 0.08	*0.57* ± *0.02*	2	1	0.47 ± 0.05	0.57 ± 0.01	0	2
Phosphatidyl serine	PS	6	0.33 ± 0.07	0.35 ± 0.01	0	0	0.29 ± 0.01	0.32 ± 0.02	0	1	0.30 ± 0.01	0.35 ± 0.01	0	1	0.35 ± 0.04	0.40 ± 0.03	0	1	0.32 ± 0.02	0.37 ± 0.01	0	1

Total levels in each lipid class are shown as nmol/mg tissue and represent mean ± SEM, with n = 4. Arrows show how many lipid species in each lipid class are either significantly decreased (↓) or increased (↑) after high-fat feeding. Significant changes (p < 0.01) after high-fat feeding are highlighted; bold highlights depict an increase while italic highlights depict a decrease after high-fat feeding.

### High-fat diet-induced changes in triacylglycerol and diacylglycerol species

**Figure 1:**
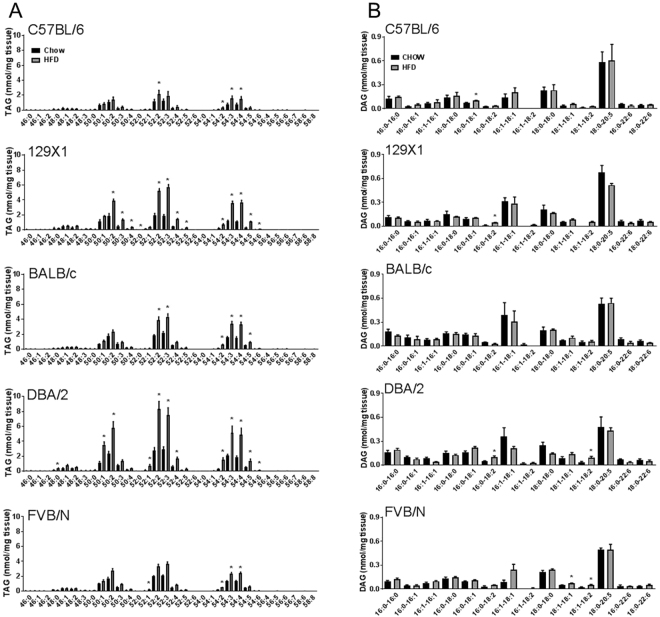
Skeletal muscle triacylglycerol (TAG) species (**A**) and diacylglycerol (DAG) species (**B**) in C57BL/6, 129X1, BALB/c, DBA/2 and FVB/N mice fed either a chow control diet (black bars) or a high-fat diet (grey bars). Shown are means ± SEM, n = 4 per group, *p < 0.01 as determined by statistical analyses outlined in Materials and Methods.

With the mass spectrometry method employed, we could not specifically distinguish the three fatty acids in each TAG species, reporting here only total fatty acyl chain length for each species, along with the number of double bonds. Relative abundance of different TAG species as well as high-fat diet induced changes were similar in the muscle of the different mouse strains, with TAG species 50:1, 50:2, 50:3, 52:2, 52:3, 52:4, as well as 54:3, 54:4 and 54:5 being the most abundant, and the most responsive to the HFD in each mouse strain (Fig. 1).

Diacylglycerol content has been associated with inhibition of insulin signalling in muscle^14^ as well as other tissues^15–17^. As mentioned above, total muscle DAG content was not affected by high-fat feeding in any mouse strain (Table 1). In line with this, we observed only minor changes in specific DAG species with high-fat feeding (Fig. 1).

### High-fat diet-induced changes in ceramide and sphingomyelin species

**Figure 2:**
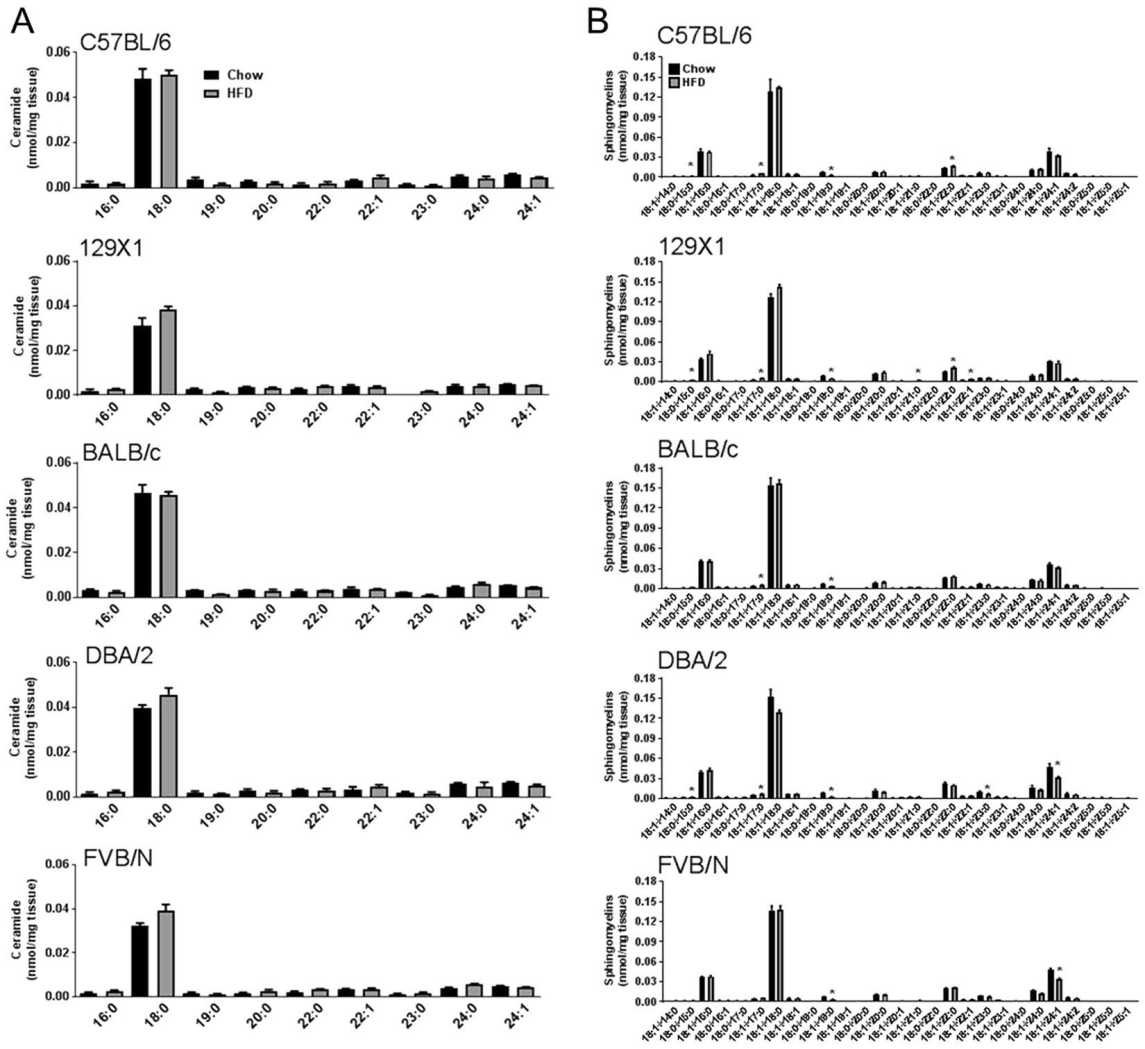
Skeletal muscle ceramide (Cer) species (**A**) and sphingomyelin species (**B**) in C57BL/6, 129X1, BALB/c, DBA/2 and FVB/N mice fed either a chow control diet (black bars) or a high-fat diet (grey bars). Shown are means ± SEM, n = 4 per group, *p < 0.01 as determined by statistical analyses outlined in Materials and Methods.

Cer 18:0, a Cer species that is associated with muscle insulin resistance^18,19^, was the most abundant Cer species in muscle, but there was no significant change in the abundance of Cer 18:0 or any other detected Cer species (Fig. 2) after high-fat feeding in any of the five mouse strains. Ceramides and SM are closely interconnected, therefore, it was not surprising that similarly SM 18:0 was the most abundant SM species in muscle across strains, followed by SM 16:0 and SM 24:1 (Fig. 2). However, similar to total SM content, we did not observe any major changes in SM species with high-fat feeding, apart from a reduction in SM 19:0 across all strains.

### High-fat diet-induced changes in muscle phospholipid composition

**Figure 3:**
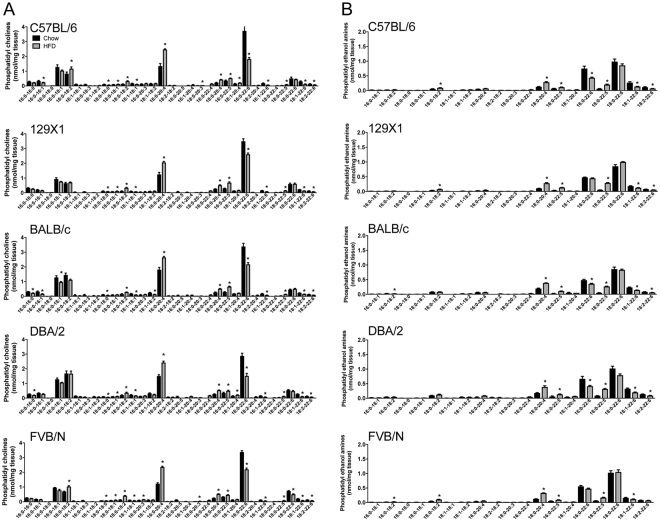
Skeletal muscle phosphatidyl choline (PC) species (**A**) and phosphatidyl ethanolamine (PE) species (**B**) in C57BL/6, 129X1, BALB/c, DBA/2 and FVB/N mice fed either a chow control diet (black bars) or a high-fat diet (grey bars). Shown are means ± SEM, n = 4 per group, *p < 0.01 as determined by statistical analyses outlined in Materials and Methods.

The most abundant PC species in muscle of all five mouse strains were PC 16:0/18:1, PC 16:0/18:2, PC 16:0/20:4 as well as PC 16:0/22:6 (Fig. 3). Interestingly, while several PC species were substantially increased with high-fat feeding in all strains (e.g. PC 16:0/20:4, PC 18:0/20:4, PC 16:0/22:5, PC 18:0/22:5), most 22:6(n-3)-containing PC species showed a significant reduction (e.g. PC 16:0/22:6, PC 16:1/22:6, PC 18:1/22:6, PC 18:2/22:6) across all strains, potentially due to the low abundance of polyunsaturated n-3 fatty acids in the HFD^20^. The most abundant PE species in muscle were PE 18:0/20:4, PE 16:0/22:6 and PE 18:0/22:6. PE 18:0/20:4 was increased with high-fat feeding in all strains, while there were more variable diet-induced alterations in PE species containing 22:6 (Fig. 3).

**Figure 4:**
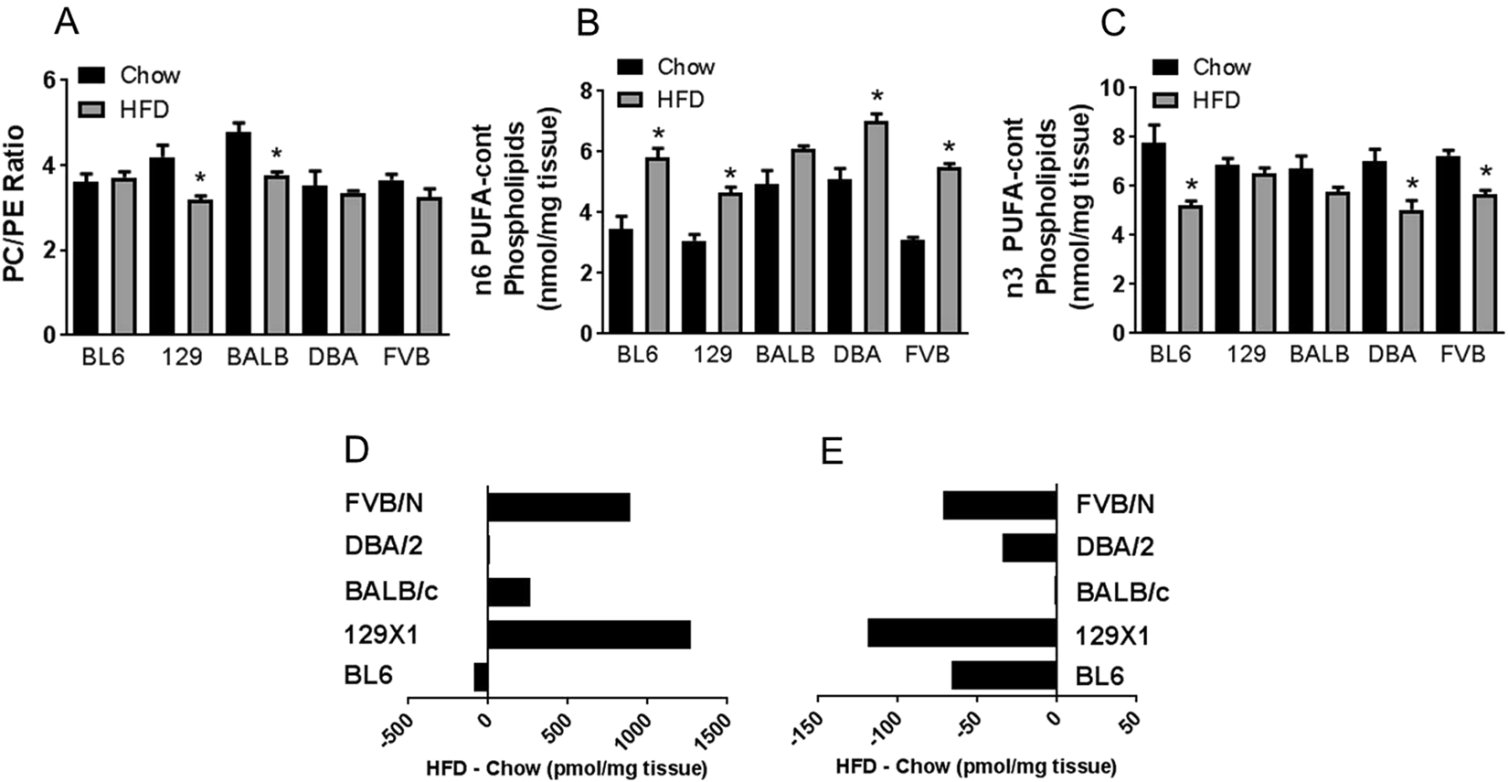
Skeletal muscle PC/PE ratio (**A**), n-6 PUFA-containing phospholipids (**B**), n-3 PUFA-containing phospholipids (**C**), as well as high-fat diet-induced changes in phospholipids containing at least one PUFA (**D**) or two PUFA (**E**) in C57BL/6, 129X1, BALB/c, DBA/2 and FVB/N mice fed either a chow control diet (black bars) or a high-fat diet (grey bars). For (D + E), results are ‘Mean HFD value − Mean CHOW value’ (n = 4 per group), with a negative value indicating a decrease and a positive value indicating an increase after HFD. For (A-C), shown are means ± SEM, n = 4 per group, *p < 0.01 as determined by statistical analyses outlined in Materials and Methods.

While metabolically active lipid intermediates such as Cer and DAG are considered the major antagonistic lipids for insulin action, there is also evidence of a role for phospholipid composition^21–24^. For example, low abundance of PE in skeletal muscle (ie. a high PC/PE ratio), especially in the membrane of the sarcoplasmic reticulum, is associated with improved insulin action and glucose uptake^22,23^. We examined the PC/PE ratio in muscle of control and HFD-fed mice (Fig. 4A) and found that the ratio was significantly decreased with fat-feeding in 129X1 and BALB/c, but remained unchanged in the other strains.

It is not only the PL class that can potentially play a role in muscle function and insulin action, but also the fatty acid composition. For example, studies have shown that increased content of polyunsaturated fatty acids (PUFA) within cell membranes increases membrane fluidity and the action of insulin^21,24^. In the five mouse strains examined, there was a relatively consistent pattern in the n-6 and n-3 PUFA across strains, with n-6 containing phospholipids increasing with HFD, while n-3 containing phospholipids were reduced (Fig. 4B,C). There was no strain-specific pattern in diet-induced changes in abundance of phospholipids containing ‘at least one PUFA’ (Fig. 4D), however high-fat feeding caused a marked decrease in PL where both fatty acids were PUFA in BL6, 129X1, DBA/2 and FVB/N mice, but not in BALB/c mice (Fig. 4E).

## Discussion

There is substantial evidence that aberrant accumulation of lipids in non-adipose tissue is strongly associated with insulin resistance, but the precise species involved and their role in different tissues is not fully resolved. The large natural variation in diet-induced metabolic deterioration across different mouse strains^1–4,25^ provides a potential avenue to pinpoint relevant lipid species with a role in insulin action. In the current study we examined if diet-induced variation in muscle lipid composition may partly explain the divergent metabolic response of BALB/c mice compared to other common inbred strains^4^. Overall we found that most changes in muscle lipid species induced by high-fat feeding were similar between BALB/c mice and other strains, suggesting that widespread changes in myocellular lipids are unlikely responsible for the favourable metabolic phenotype in BALB/c mice.

Consistent with many previous studies^25–28^, we observed a robust increase in TAG levels in the muscle of all mouse strains in response to high-fat feeding. TAGs are considered to be largely inert and other more bioactive lipid intermediates are thought to play a more causal role in insulin resistance. For the past 2 decades, evidence has accumulated in human and animal studies implicating Cer in the generation of insulin resistance^29–34^. While there is evidence that pharmacological reduction of total Cer synthesis can have beneficial metabolic outcomes^35,36^, until recently most sphingolipid species were thought to have similar metabolic effects. It is now clear, however, that Cer and SM species vary between tissues, and have distinct effects on metabolic processes^37–40^. There was a divergent pattern in the hepatic sphingolipid profile of mice from this study, with the favourable metabolic phenotype of BALB/c mice associated with an altered balance of long chain (e.g. C16:0) vs. very long chain (e.g. C24:0) sphingolipids^8^. In skeletal muscle, C18:0 is the dominant form of Cer, accounting for ~60–80% of the total. Studies in humans have linked C18:0 with insulin resistance^18,19^ and in muscle of fat-fed mice, accumulation of ‘long-chain’ Cer, especially Cer16:0 and Cer18:0, has been shown to be associated with the development of insulin resistance^26,41^. Across the strains in the current study muscle Cer were not strongly associated with insulin resistance induced by our high-fat diet.

Besides Cer, accumulation of diacylglycerols has been previously associated with insulin resistance in both rodents and humans, particularly hepatic insulin resistance^16,42^. Diacylglycerols, accumulate in the insulin resistant state^26,43–45^, and are thought to activate protein kinase C (PKC) isoforms that inhibit insulin receptor substrate 1 (IRS1), thus blocking insulin action^42,46^. The total DAG levels, as well as specific DAG species, were essentially unchanged by the HFD in our study and could not explain the disparate metabolic response of BALB/c mice. There are a number of other models where insulin resistance develops independently of changes in DAG levels^35,47^, and a recent study where an enzyme involved in phosphatidylethanolamine synthesis was disrupted in muscle, showed marked increases in muscle DAG without any change in insulin sensitivity^48^. One caveat to our study and many others is that the subcellular distribution of different lipids has not been measured. It is possible that localised changes in specific lipids (e.g. Cer, DAG) may be more important for regulating insulin action and metabolic function than changes in bulk content, as has been suggested for DAG in other studies^49–51^.

In addition to changes in the levels of active lipid intermediates (ie. Cer and DAG), membrane fatty acid composition of skeletal muscle has been linked with the regulation of glucose metabolism and insulin action^21,24^. The physicochemical properties of a membrane bilayer are to a large extent determined by the nature of the fatty acids present in that membrane, with more polyunsaturated fatty acids being associated with increased membrane fluidity, increased number of insulin receptors, and an improvement in the action of insulin^21,52–55^. Here we showed that mouse strains that are susceptible to diet-induced insulin resistance (BL6, 129X1, DBA/2 and FBB/N) all exhibit a HFD-induced reduction in phospholipids that contain a PUFA in both the sn1 and sn2 position, whereas this reduction was not observed in the insulin-sensitive BALB/c mice. Differential PL acylation could be potentially related to strain-specific differences in the biosynthesis of new PL (e.g. in the Kennedy pathway) or through altered dynamics of PUFA incorporation during acyl remodelling of the muscle membrane bilayer in the Lands cycle^56^. It should be noted however, that the number of double-PUFA phospholipids was only a small fraction of the total phospholipid pool (1.0–2.6%), so the relevance of these changes to the phenotype of BALB/c mice is unclear. However it is interesting to note that there were no strain-specific differences with regards to phospholipids containing a PUFA in either the sn1 or sn2 position (ie. only one of both FA being polyunsaturated, most commonly on the sn2 position). Two polyunsaturated fatty acids in close proximity are more likely to increase membrane fluidity as the ‘kinks’ resulting from the C-C double-bonds makes them pack less tightly^54^, and are therefore likely to have a greater impact on membrane protein fluidity and activity^57^. Given that the dynamic nature of the membrane can influence insulin receptor localisation and signalling^21,24,58–60^, further studies are needed to determine the role of double-PUFA phospholipids in influencing insulin sensitivity in muscle and other tissues.

Among polyunsaturated fatty acids there is also evidence that n-3 PUFA may play a favourable role in improving insulin action; certainly a high n-6/n-3 ratio appears deleterious^24,61^. In rodents and humans, it was shown that a n-3 PUFA-enriched diet increased content of n-3 PUFA in membrane lipids (while simultaneously decreasing n-6 PUFA content) and improved insulin action in skeletal muscle, as well as other tissues^62–65^. In this study, there was a general trend for an accretion of n-6 PUFA-containing phospholipids at the expense of n-3 PUFA-containing phospholipids in response to the HFD. Interestingly, BALB/c mice displayed the smallest increase in n-6 PUFA-containing phospholipids and only a minor decrease in n-3 PUFA-containing phospholipids, indicating some resistance to high-fat diet-induced membrane PUFA remodelling in this strain.

While the phospholipid fatty acid composition plays a major role in determining membrane fluidity, phospholipid head groups also play an important role in membrane integrity^66^. A reduction in phosphatidylethanolamine (an increased PC/PE ratio), has been associated with improved insulin action in skeletal muscle^22^, potentially due to changes in sarcoplasmic reticulum SERCA activity, increasing cytosolic calcium and activating calcium- and AMPK-dependent pathways^67^. High-fat feeding decreased the PC/PE ratio in skeletal muscle of all mouse strains in the current study, including BALB/c mice, indicating it is unlikely a major determinant of muscle insulin sensitivity under our experimental conditions.

In conclusion, our extensive comparison suggests that unlike other tissues^8^, the lipid profile of skeletal muscle responds in a generally uniform manner to a high fat diet across different mouse strains. The relative protection against diet-induced defects in glucose homeostasis and insulin action in BALB/c mice^4^ could not be explained by differential changes in the total amount of lipid intermediates generally linked with insulin resistance (e.g. DAG, Cer), with the only divergent response being a lack of diet-induced losses of double-PUFA containing phospholipids. The contrast of the lipid changes in muscle with those observed in the liver of these same animals^8^, highlights the independent regulation of lipid metabolism between different tissues and suggests that multiple mechanisms may contribute to the variable metabolic profile observed between different mouse strains.

## Materials and Methods

Eight-week old male C57BL/6 J, 129X1/SvJ, BALB/c, DBA/2 and FVB/N mice were purchased from the Australian Resource Centre (Perth, WA, Australia). Mice were maintained in a temperature-controlled room (22 °C ± 1 °C) with a 12-hour light/dark cycle and *ad libitum* access to food and water. After one week on a standard low-fat chow diet (CHOW), mice were randomly allocated to remain on the CHOW or to receive a high-fat diet (HFD) *ad libitum* for 8 weeks (detailed diet composition can be found in^4^). Tissues were collected from mice at 9–10 am without any prior fasting period. All experiments were approved by the Garvan Institute/St. Vincent’s Hospital Animal Experimentation Ethics Committee and followed guidelines issued by the National Health and Medical Research Council of Australia. Detailed methodology of the metabolic characterization of these mice can be found in our recent paper^4^.

### Lipid extraction

Lipid extraction was performed according to the method of Matyash *et al*.^68^. In brief, 25 mg of frozen muscle tissue was added to 2 mL tough tubes (Geneworks, Hindmarsh, SA, Australia) on dry ice and stored at −80 °C. An aliquot of 200 µl of methanol containing 0.01% butylated hydroxytoluene and internal standards (please refer to our recent manuscript for a detailed description of internal standards use^8^); were added to each tube. Samples were homogenised on a FastPrep-24 (MP Biomedical, Seven Hills, NSW, Australia) at 6 m/s for 40 sec. Beads were washed with 200 µl methanol and the wash added to homogenate. Aliquots of 300 µl were removed to 2 ml microcentrifuge tubes (Eppendorf, North Ryde, NSW, Australia), 1 ml of methyl-tert butyl ether was added and samples were vortexed for 1 hr at 4 °C. An addition of 250 µl of 150 mM ammonium acetate (Fluka LC-MS grade) was added to induce phase separation. Tubes were vortexed and spun at 2000 g for 5 minutes to complete phase separation. Approximately 800 µl of the upper organic layer was removed to a new 2 ml glass vial, dried under a stream of nitrogen at 37 °C, resuspended in 500 µl methanol:chloroform (2:1 v/v) and stored at −20 °C until analysis. Samples were diluted 100-fold into methanol:chloroform (2:1 v/v) containing 5 mM ammonium acetate prior to mass spectrometric analysis. An aliquot of each extract was hydrolyzed to remove acyl-linked lipids and re-extracted and improve mass spectrometric analysis of spingolipids. 200 µl of extract was added to 185 µl of methanol containing 0.01% butylated hydroxytoluene, 27 µl of 10 M NaOH was added (final concentration 0.7 M), and gently tumbled for 2 hr at room temperature. 615 µl of MTBE was added, followed by 200 µl of aqueous ammonium acetate to induced phase separation. Tubes were vortexed and spun at 2000 g for 5 minutes to complete phase separation. The upper organic layer was removed to a new 2 ml glass vial, and diluted 200-fold into methanol:chloroform (2:1 v/v) containing 5 mM ammonium acetate prior to mass spectrometric analysis.

### Mass Spectrometry

Mass spectra were acquired using a chip based nano-electrospray ionization source (TriVersa Nanomate®, Advion, Ithaca, NY, USA) coupled to a hybrid linear ion trap-triple quadrupole mass spectrometer (QTRAP® 5500, ABSCIEX, Foster City, CA, USA). 10 µl of extract was aspirated from a sealed 96-well plate and delivered into the mass spectrometer via a nano- electrospray ionization (ESI) chip with an orifice diameter of 4.1 µm. The delivery gas was N2 at a pressure of 0.4 psi and a spray voltage of 1.1 kV and −1.1 kV was used for positive and negative ion acquisition, respectively. For target lipids and MS scan parameters please refer to our recent manuscript^8^. Experimental conditions for positive ion mode acquisition were a declustering potential of 100 V, entrance potential of 10 V and a scan rate of 200 m/z units.s-1. Negative ion mode acquisition parameters were a declustering potential of −100 V, entrance potential −10 V and scan rate 200 m/z units.s-1. Mass spectra were averaged over 30 scans in positive mode and 40 scans in negative mode. Data were analyzed with LipidView® (ABSCIEX) software version 1.2, including smoothing, identification, removal of isotope contribution from lower mass species, and correction for isotope distribution. Ionized lipids detected with a signal-to-noise ratio (s/n) over 10 were included in the analysis. Quantification was achieved in LipidView® software by comparison of the peak area of individual lipids to their class-specific internal standards after isotope correction. To obtain quantification of the molecular species of phospholipids, absolute quantification of phsophatidylcholine (PC), phosphatidyletanolamine (PE), and phsosphatidylserine (PS) species was first obtained by comparison to internal standards in head-group scans in positive mode. Next, negative ion mode precursor ion scanning for fatty acyl chains was used to determine respective fatty acyl chains for each lipid species. Ions detected at masses that could be assigned to ether-linked or odd-chain fatty acids were assumed to be ether-linked species. In cases where more than one isobaric species was detected in a class by negative fatty acyl scans, the fractional intensities of each isobaric species was applied to the absolute quantification obtained in positive mode head group scans. Ether-linked PE species do not produce the 141 head group fragment as efficiently as the di-acyl PE species; therefore a correction factor of 3.45 was applied to all ether-PE species^69^. Sphingomyelin species were detected using the 184.1 m/z precursor ion scan which cannot distinguish between isobaric DHSM and SM species. The SM species are assumed to contain the major sphingolipid backbone d18:1, and are listed as species indicating the respective N-linked fatty acid. Lipid species are notated in accordance with recently proposed shorthand by Liebisch *et al*.^70^.

### Statistical Analysis

Total levels of the various lipid classes are presented as mean ± SEM. Log-transforms of the lipidomics data were taken to ensure normal distributions prior to the statistical analyses. Significant differences in the lipid expression between CHOW and HFD in the different strains were assessed via a one-way ANOVA. The p-values of the ANOVA analyses for all the lipid species were then ranked and the normal probability plots examined to determine statistical significance. It was observed that the p-value versus rank was highly non-linear, in particular for data with P < 0.05 (not shown), indicative of statistically significant results. For only those lipids with ANOVA P < 0.05, post-hoc testing was undertaken to determine the species which had significant differences between CHOW and HFD, using a Protected Fisher’s Least Significant Difference procedure with p < 0.01. Statistical analyses were performed using custom scripts in Matlab 2016b (Mathworks).

### Data Availability

Raw data files supporting the results and conclusions presented in this manuscript are available from the corresponding author upon reasonable request.
